# Model Optimization for the Prediction of Red Wine Phenolic Compounds Using Ultraviolet–Visible Spectra

**DOI:** 10.3390/molecules25071576

**Published:** 2020-03-30

**Authors:** Chris Beaver, Thomas S Collins, James Harbertson

**Affiliations:** Viticulture and Enology Program, Washington State University Tri-Cities, 2710 Crimson Way, Richland, WA 99354, USA; cwb13@wsu.edu (C.B.); tom.collins@wsu.edu (T.S.C.)

**Keywords:** mathematical modeling, red wine phenolics, UV–vis spectroscopy

## Abstract

The primary objective of this work was to optimize red wine phenolic prediction with models built from wine ultraviolet–visible absorbance spectra. Three major obstacles were addressed to achieve this, namely algorithm selection, spectral multicollinearity, and phenolic evolution over time. For algorithm selection, support vector regression, kernel ridge regression, and kernel partial least squares regression were compared. For multicollinearity, the spectrum of malvidin chloride was used as an external standard for spectral adjustment. For phenolic evolution, spectral data were collected during fermentation as well as once a week for four weeks after fermentation had ended. Support vector regression gave the most accurate predictions among the three algorithms tested. Additionally, malvidin chloride proved a useful standard for phenolic spectral transformation and isolation. As for phenolic evolution, models needed to be calibrated and validated throughout the aging process to ensure predictive accuracy. In short, red wine phenolic prediction by the models built in this work can be realistically achieved, although periodic model re-calibration and expansion from data obtained using known phenolic assays is recommended to maintain model accuracy.

## 1. Introduction

The phenolic content of wines produced form *V. vinifera* berries can vary widely for several reasons, including vineyard practices [1], cultivar [2,3], vineyard geography [3,4], vintage [5], and wine making practices [6]. Phenolic quantitation is invaluable from a commercial perspective, particularly for red wines that have a greater and more diverse phenolic content than wines made from white cultivars [7] due to the duration of skin contact during red wine production [6].

As wine phenolics possess similar chemical structures, they also possess similar ultraviolet–visible (UV–Vis) spectra. For this reason, several methods aimed at isolating wine phenolics by class have been developed [8,9,10,11,12]. Analysis of phenolics using HPLC and mass spectrometry has also been developed [13]. Regardless of the methodology, phenolic analysis by separation is consumptive of time and resources to obtain accurate results. For that reason, several researchers have attempted to circumvent this necessity by implementing multivariate statistical analysis.

Modern statistical learning theory began in the 1960s with Rosenblatt’s perceptron [14]. Since that time, the development of modern computers has permitted highly accurate methods for identification [15], classification [16], and prediction [17] across many fields, including enology. For example, Skogerson et al. [18] applied partial least squares regression (PLSR) to predict the phenolic composition of wine during fermentation from its UV–Vis spectra. Beyond phenolic prediction, Hosu et al. [19] predicted the antioxidant capacity in Romanian red wines using UV–Vis spectroscopy and artificial neural networks. As for alcohol and titratable acidity (TA), Yu et al. [20] used a least squares support vector machine (LS-SVM) to accurately predict the alcohol content and TA in Chinese rice wine by recording the wine’s UV–Vis and near-infrared spectrum (350 nm–1200 nm). Sensorial predictive models have also been constructed. Lombardo and Veaux [21] proposed a nonlinear application of PLSR using Multivariate adaptive regression splines (MARS) for the sensorial analysis of both red and white wines.

While modern machine learning approaches have been successfully applied in various ways to enological analysis, the application of such techniques remains experimental. This study attempted to measure the validity of phenolic model prediction in three steps:

1. Compare several multivariate regression models to determine which gives the most accurate predictions for wine phenolics (tannins, anthocyanins, and total iron reactive phenolics).

2. Address phenolic multicollinearity in the UV–Vis spectra by mathematically isolating individual phenolics through the spectrum of a malvidin chloride standard.

3. Compare the final adapted phenolic model predictions across two vintages and two instruments.

## 2. Results and Discussion

### 2.1. Algorithm Comparison and Overall Performance

Table 1 compares the performance of the three algorithms used for phenolic prediction. The first three rows are for anthocyanins, rows 4 through 6 are for tannins, and rows 7 through 9 are for total iron reactive phenolics (TIPs). All root mean squared error values were calculated by taking the square root of the squared sum difference between predicted values and observed values divided by the number of observations (Equation (1)).

**Equation (1):** Root mean squared error (RMSE) equation.

*RMSE* = √∑(*P* − *O*)^2^/*N* (1)

where *P* is equal to predicted values, *O* is equal to observed values, and *N* is equal to the number of observations.

Root mean squared errors of calibration (RMSEC) in some cases were smaller than that of root mean squared errors of prediction and cross-validation (RMSEP and RMSECV), while the R^2^ values for prediction and cross-validation (R^2^*_P_* and R^2^*_CV_*) were generally larger than R^2^ values for calibration (R^2^*_C_*). In these cases, the RMSEC was always smaller than the RMSEP regardless of cost, so these sets were optimized by choosing the cost that maximized the R^2^*_P_*. Support vector regression (SVR) outperformed the other two algorithms overall.

The initial model for this project was built from data acquired using a single spectrophotometer from a single vintage. While the model calibrated and validated well, new predictions made were quite poor as the spectrophotometer available was different from that used in the original work. Beyond different spectrophotometers, the vintage and the grape-growing regions were also different in the new data set, unlike previous work which utilized a single fruit source and vintage [18,22]. This was addressed in three steps. The first two steps addressed spectral multicollinearity issues independent of the instrument in use, and the third step addressed the different instrumentation issues.

### 2.2. Spectral Multicollinearity

In the UV–Vis absorbance spectra of red wine, the spectra of several phenolics overlap including the ones measured here. This can be problematic in building predictive models as it becomes difficult to determine exactly how much absorbance at a given wavelength in the spectra is due to a particular phenolic compound or compound class. For assay measurement of phenolics by UV–visible absorbance, the compounds of interest are typically isolated chemically before the final absorbance is recorded [12,23]. Anthocyanins, for example, can be isolated by dropping the pH [24]. Tannins can be isolated through protein precipitation [25], while polymeric pigment isolation can be accomplished through bisulfite bleaching [26]. A goal of this work was to eliminate or at least minimize the need for chemical isolation of phenolics. To achieve this, the spectra for individual phenolics were isolated mathematically. For anthocyanins, this was easily achieved by only considering the visible spectra (430 nm–700 nm) to make predictions as TIPs and tannins have no absorbance in the visible range. For TIPs and tannins, absorbance in the UV range of the spectra (230 nm–429 nm) due to the presence of anthocyanins had to first be estimated and removed. To calculate this estimate, the entire spectrum of the malvidin chloride (MC) standard was transformed such that the absorbance at each wavelength was a percentage of the sum total, such that the spectrum summed to one. Next, the portion of each spectrum in the raw data due to anthocyanins below 430 nm was estimated by multiplying each wavelength in the transformed MC spectrum below 430 nm by the raw wine spectra at 520 nm divided by the transformed MC spectra at 520 nm (Equation (2)). Lastly, each calculated anthocyanin spectrum below 430 nm was subtracted from each raw wine spectrum below 430 nm to give the final spectra for TIPs and tannins.

**Equation (2):** The phenolic spectra used to predict tannins and total iron reactive phenolics (TIPs) was generated by multiplying each point in the transformed malvidin chloride (MC) spectra below 430 nm by the raw sample spectra at 520 nm divided by the MC spectra at 520 nm.

For *i* rows and *j* columns in each spectrum@1:430 nm:

Transformed Spectra*_i_*_,*j*_ = MC*_j_* × (Raw spectra@520nm*_i_* /MC@520 nm*_i_*) (2)

It is important to emphasize that the predictive models presented are meant to predict the chemical phenolic composition of a given red wine only rather than its perceived sensorial aspects [27]. While the sensorial perception of a wine is obviously important, building such a model is beyond the scope of this work.

### 2.3. Instrumentation

In an ideal world, every UV–Vis absorbance spectrophotometer would be identical in every way. This is of course not the case but having the ability to apply the same predictive model across different instruments would be advantageous. For that reason, two different instruments were compared in this study, namely the Genesys 10S produced by Thermo Fisher Scientific (Waltham, MA) and the Cary 14 spectrophotometer produced by Olis (Bogart, GA) to address this issue. The two instruments differed in several areas, including instrument sensitivity, absorbance quantification range, and available spectral range. The first data set compared several different dilutions for data acquired using the Genesys 10S spectrophotometer. Once the optimal dilution for that instrument was determined, a new sample set was acquired from a new vintage and a different region. Several different ratios of model wine to wine were tested in the Cary 14 spectrophotometer until the scaled spectra of the new samples closely resembled the average of the scaled spectra from the Genesys S10 data set. The difference in optimal dilutions between the two spectrophotometers was considerable (a 1:5 dilution was optimal for the Genesys S10, 1:25 was optimal for the Cary 14).

Unfortunately, simply calibrating an instrument using an accepted standard is not a reliable way to apply a multivariate predictive model across different instruments. Beyond absorbance sensitivity (spectral resolution), other variations such as signal to noise ratio and ultraviolet absorbance to visible absorbance ratio can and do vary between instruments. For this reason, whenever a predictive model is implemented with a new instrument, it is strongly recommended that a subset of data using the new instrument be added to the original data set. The subset should contain both assay data and the concomitant spectral data. The combined data set should then be calibrated and validated to maximize model predictive accuracy using the new instrument.

### 2.4. Phenolic Evolution

Polymeric pigments are formed through reactions of tannins, other phenolics, and keto-acids with anthocyanins [6,11,12,28]. The spectral data acquired in this study suggests a significant change in color occurred within the first month after fermentation was complete. Table 2 shows that correlations between phenolic assay measurements and the respective absorbance values of the wine at 520 nm and 280 nm fluctuated greatly over time. Table 3 demonstrates that by the fourth week, there was a significant negative correlation between anthocyanins and TIPs as well as anthocyanins and tannins.

While spectral transformation did greatly improve predictive power for tannins and TIPs, there remained a certain level of inherent error for tannins and TIPs as there is no wavelength in the UV spectra in which tannins and TIPs do not overlap. TIPs are very heterogeneous by nature, and for this reason, there are no established external standards available for TIPs. This makes spectral isolation of tannins and TIPs difficult if not impossible. Despite this, tannin and TIP models performed well, with root mean squared error values below ten percent. This suggests that spectral transformation by removing the calculated malvidin chloride spectra was enough to generate trustworthy tannin and TIP spectra, so long that the model was re-calibrated by combining the old data set with some new data.

Just as with tannins and TIPs, polymeric pigment formation institutes a significant source of predictive error as the formation of such pigments significantly changes the overall correlation between the assay data with any given point in the spectra. For example, while fermenting wines had the highest correlation with measured anthocyanins at 524 nm (0.87), wines four weeks after fermentation was complete had the highest correlation with measured anthocyanins at 357 nm (0.82). Unfortunately, the model applied in this study did not calibrate for polymeric pigments, although it is difficult to say how accurate a predictive model for polymeric pigments built using UV–Vis spectroscopy could be. As mentioned, the spectra of tannins and TIPs overlap, which presents an inherent source of error in tannin and TIP prediction. Polymeric pigments represent a very heterogeneous group of compounds that could be formed not only from covalent interactions between tannins and anthocyanins but also through such interactions between tannins and TIPs, or tannins, anthocyanins, and TIPs. While tannin and TIP models can be adjusted by mathematically removing the estimated spectra of malvidin chloride, an accurate adjustment is difficult for polymeric pigments due to the heterogeneity of the class and, therefore, the heterogeneity of the spectra. Phenolic oxidation over time only further adds to the complexity of such a model. When considering all of these factors together, it becomes more apparent as to why there are no obvious trends among the correlation values depicted in Table 1 and Table 2.

## 3. Materials and Methods

### 3.1. Instrumentation

UV–Vis spectra from 230–700 nm were collected in 1 nm increments using a Genesys 10S UV–Vis spectrophotometer (Thermo Scientific, Waltham, MA, USA) Samples were diluted as necessary using model wine to obtain an absorbance of less than 2.0 absorbance units at 230 nm. Model wine was produced by combining 120 mL of 90 proof ethanol with 880 mL of deionized water and adjusting the pH to 3.3 using 0.1 N HCl. Tannin, anthocyanin, and total iron reactive phenolic measurements were done on all samples according to the methods of Harbertson and Spayd [12].

### 3.2. Sample Collection and Analysis

For model construction, spectral and assay data collected at a commercial facility in Napa Valley, CA during the 2010 vintage was combined with spectral and assay data collected from a university facility in Richland, WA during the 2016 vintage for a total of 323 samples. Samples were collected daily throughout fermentation. Fermenting samples were sterile filtered and divided. One portion of each sample was used to conduct UV–visible assays for tannins [25], anthocyanins, and iron reactive phenols [5]. The remaining portion of each sample was used for collection of the entire UV–Vis spectrum from 230 nm to 700 nm at one nanometer increments of the sample.

To track phenolic evolution over time, 45 samples were collected from a university facility in Richland, WA in one-week intervals starting immediately after fermentation ended during the 2016 vintage. UV–visible assays for anthocyanins [5], tannins [25], and total iron reactive phenolics [5] were conducted on a portion of each sample. The remaining portion of each sample was used to gather data for the entire UV–Vis spectrum from 230 nm to 700 nm in one nanometer increments.

### 3.3. Model Comparison

Three regression algorithms were compared: support vector regression (SVR) [29], kernel ridge regression (KRR) [30], and kernel partial least squares regression (KPLSR) [31]. Regardless of the method, an optimal weight value was determined to ensure maximum model performance: for SVR, the value was the cost function, for KPLSR, it was the number of components, and for KRR, it was the alpha term. For cross-validation, each data set was randomized and then divided into two subsets. Approximately 90% of the total set was treated as the training set, and the remaining 10% was treated as the test set. The training set was used to calibrate the model by comparing predicted values with the measured assay values being careful not to overfit the model. Once built, the model was used to make predictions using the spectral data alone of the corresponding test set. This process was repeated ten times. For algorithms where there was no minimum weight limit (SVR, KRR), the minimum weight was set when the coefficient of determination for measured versus predicted concentrations of the phenolic in question was less than 0.5. The weight value was then systematically increased until the coefficient of determination for the test sets reached a maximum and began to decrease. The optimal weight for each algorithm was chosen to be that which gave the most accurate predictions when a new spectral data was applied. This process was repeated for each phenolic class tested.

### 3.4. Software

All data analysis and plotting were conducted using the R project for statistical computing. For SVR, the e1071 package version 1.7-2 [32] was used, for KRR, the glmnet version 3.0-2 [33] and chemometrics version 1.4.2 [34] packages were used, for PLSR, the pls package version 2.7-2 [35] was used, and for cross validation, the caret [36] package was used.

## 4. Conclusions

Phenolic prediction by recording the entire UV–Vis spectra of wine can realistically be achieved if models are periodically updated to account for phenolic evolution over time. Furthermore, different spectrophotometers can be used to make predictions using the same model if a small subset of calibration samples is added to the model. From the three algorithms compared, support vector regression gave the most accurate predictions. In closing, while this work did demonstrate what is needed to be done to obtain and maintain reliable phenolic predictions for young wines from UV–Vis spectral data, a clear-cut method to obtain reliable phenolic predictions for aging wines without the need for assay data remains elusive. It may be necessary to calibrate models over the entire lifespan of a wine set to realistically construct assay free models but the amount of work this constitutes is very small compared to the reference analysis.

## Figures and Tables

**Table 1:** Comparison of the predictive performance of support vector regression (SVR), kernel ridge regression (KRR), and kernel partial least squares regression (KPLSR) for the prediction of anthocyanins, tannins, and total iron reactive phenolics (TIPs) in red wine. RMSEC is the root mean squared error of calibration, RMSEP is the root mean squared error of prediction, and RMSECV is the root mean squared error of cross-validation. R^2^*_C_* gives R^2^ values for calibration, R^2^*_P_* gives R^2^ values after for prediction, and R^2^*_CV_* gives R^2^ values for cross-validation.

Phenolic Algorithm	R^2^*_C_*	RMSEC	R^2^*_P_*	RMSEP	R^2^*_cv_*	RMSECV
Anthocyanins*_SVR_*	0.84	55.27	0.87	57.80	0.96	43.69
Anthocyanins*_KRR_*	0.87	48.61	0.87	54.99	0.91	54.34
Anthocyanins*_KPLSR_*	0.84	54.47	0.89	50.43	0.94	49.77
Tannins*_SVR_*	0.91	98.06	0.94	94.18	0.97	68.70
Tannins*_KRR_*	0.92	97.80	0.94	97.55	0.95	105.68
Tannins*_KPLSR_*	0.84	124.20	0.90	121.45	0.97	77.84
TIPs*_SVR_*	0.88	217.55	0.92	215.47	0.94	219.33
TIPs*_KRR_*	0.92	186.73	0.92	219.26	0.93	225.07
TIPs*_KPLSR_*	0.87	218.71	0.90	237.79	0.90	228.00

**Table 2:** Comparison of correlations between values obtained by assay and that same wine’s absorbance values at 520 nm as well as 280 nm at different time points after fermentation was complete. Assay data used to calculate correlation coefficients was a subset of that used for modeling (n = 44).

Phenolic ID	Initial	Week 1	Week 2	Week 3	Week 4
Anthos @520	0.75	0.04	0.80	−0.03	−0.04
TIPs@520	0.71	−0.59	0.54	0.54	0.22
Tannin@520	0.04	0.44	0.47	0.36	0.11
Anthos@280	0.45	0.57	0.63	0.04	0.44
TIPs@280	0.73	0.89	0.94	0.61	0.36
Tannin@280	0.43	0.89	0.94	0.54	0.34

**Table 3:** Comparison of correlations between values obtained by assay between anthocyanins and total iron reactive phenols (TIPs) as well as between anthocyanins and tannins. By the fourth week, there was a significant negative correlation between anthocyanins and the other two phenolics and between anthocyanins and tannins, suggesting pigmentation. Assay data used to calculate correlation coefficients were a subset of that used for modeling (n = 44).

Phenolic ID	Initial	Week 1	Week 2	Week 3	Week 4
TIPs	0.69	0.33	0.51	0.01	−0.63
Tannins	0.03	0.19	0.44	0.10	−0.61
